# Short-interval traffic lines: versatile tools for genetic analysis in *Arabidopsis thaliana*

**DOI:** 10.1093/g3journal/jkac202

**Published:** 2022-08-26

**Authors:** R Scott Poethig, William L Cullina, Erin Doody, Taré Floyd, Jim P Fouracre, Tieqiang Hu, Mingli Xu, Jianfei Zhao

**Affiliations:** Department of Biology, University of Pennsylvania, Philadelphia, PA 19146, USA; Department of Biology, University of Pennsylvania, Philadelphia, PA 19146, USA; Department of Biology, University of Pennsylvania, Philadelphia, PA 19146, USA; Department of Biology, University of Pennsylvania, Philadelphia, PA 19146, USA; Department of Biology, University of Pennsylvania, Philadelphia, PA 19146, USA; Department of Biology, University of Pennsylvania, Philadelphia, PA 19146, USA; Department of Biology, University of Pennsylvania, Philadelphia, PA 19146, USA; Department of Biological Sciences, University of South Carolina, Charlottesville, SC 29208, USA; Department of Biology, University of Pennsylvania, Philadelphia, PA 19146, USA

**Keywords:** balancer chromosome, fluorescent-tagged lines, recombination, *myb75*, *fie*, *val1*, Plant Genetics and Genomics

## Abstract

Traffic lines are transgenic stocks of *Arabidopsis thaliana* that contain a pair of linked seed-specific eGFP and DsRed markers. These stocks were originally developed for the purpose of studying recombination, but can also be used to follow the inheritance of unmarked chromosomes placed in *trans* to the marked chromosome. They are particularly useful for this latter purpose if the distance between markers is short, making double recombination within this interval relatively rare. We generated 163 traffic lines that cover the *Arabidopsis* genome in overlapping intervals of approximately 1.2 Mb (6.9 cM). These stocks make it possible to predict the genotype of a plant based on its seed fluorescence (or lack thereof) and facilitate many experiments in genetic analysis that are difficult, tedious, or expensive to perform using current techniques. Here, we show how these lines enable a phenotypic analysis of alleles with weak or variable phenotypes, genetic mapping of novel mutations, introducing transgenes into a lethal or sterile genetic background, and separating closely linked mutations.

## Introduction

Identifying a mutation or polymorphism of interest in the progeny of a genetic cross is central to genetic analysis. If the molecular identity or chromosomal location of the mutation/polymorphism is known, this can be done using molecular methods. However, these methods are destructive and time-consuming and require resources that may not be available in some circumstances. We developed a set of transgenic lines of *Arabidopsis thaliana* that make it possible to visually determine the genotype of seeds in a segregating population prior to planting (Wu *et al.* 2015). Each transgenic line—termed a “Traffic Line” (TL) because of its green, red, and yellow phenotypes—possesses a pair of linked *pNAP::eGFP* and *pNAP::DsRed* transgenes. The reporters in these transgenes are under the regulation of the seed-specific Napin promoter and thus produce, respectively, green or red fluorescent seeds (Stuitje *et al.* 2003). Such lines were originally developed for the purpose of studying recombination (Melamed-Bessudo *et al.* 2005), but also make it possible to follow the transmission of the chromosome segment that is in *trans* to marked segment (Wu *et al.* 2015). Indeed, a TL can be used much like a balancer chromosome (Casso *et al.* 2000; Hentges and Justice 2004) to follow the segregation of genes located in a defined region of the genome.

Recombination between the transgenes in a TL is useful if the goal is to study factors that influence recombination (Melamed-Bessudo and Levy 2012; Ziolkowski *et al.* 2015, 2017; Saini *et al.* 2020; Nageswaran *et al.* 2021; Kbiri *et al.* 2022). However, recombination is disadvantageous if the TL is being used to follow the segregation of an unmarked chromosome segment in *trans* to the TL because double recombination within the interval generates an unmarked chromosome that may be genetically different from the original unmarked chromosome. Recombination is minimized if the interval between the markers in a TL is relatively short. Such “short-interval” TLs are not only useful as balancers but also facilitate experiments that rely on the ability to detect recombination in small, defined chromosomal regions. These include studies of the factors that influence recombination frequency, removing second-site mutations/polymorphisms linked to a gene of interest, and fine mapping a QTL or a newly induced mutation known only by its phenotype.

Our initial set of TLs contained transgenes located an average of 4.8 Mb (21 cM) from each other (Wu *et al.* 2015). Here, we describe a collection of 163 TLs that covers the *Arabidopsis* genome in much shorter overlapping segments. In addition, we present examples of how TLs facilitate genetic analysis by providing a rapid, visual method for determining the genotype of plants in a segregating population.

## Materials and methods

### Plant material

The recombinant lines described here are in the Columbia (Col) accession and were produced from transgenic lines containing sequenced insertions of *pNAP::eGFP* and *pNAP::DsRed*, as described previously (Wu *et al.* 2015).

### Microscopy

Dry seeds were observed with a Leica MZ FLIII fluorescent stereomicroscope, using long-bandpass GFP (excitation 480/40 nm, barrier 510 nm), short-bandpass GFP (excitation 470/40 nm, barrier 525/50 nm), and DsRed (excitation 546/12 nm, barrier ET560 nm) filter sets. The long-band pass filter was particularly useful because it transmits enough red light to enable simultaneous scoring of GFP and DsRed fluorescence.

### Producing a TL

Plants containing a *pNAP::eGFP* insertion were intercrossed with a plant containing a linked *pNAP::DsRed* insertion, and the progeny of this cross were then crossed as pollen parents to Col. Progeny from this cross were scored for red fluorescent, green fluorescent, nonfluorescent, and green and red fluorescent seed to measure the recombination distance between the 2 transgenes; seeds that expressed both green and red fluorescence were planted and allowed to self-pollinate. Self-pollinated seeds displayed varying intensities of green, red, and green and red fluorescence. Seeds with the strongest green and red fluorescence were planted to select for plants homozygous for the recombinant *pNAP::eGFP* and *pNAP::DsRed* chromosome, and seed stocks were established from these homozygous plants.

### Construction of *FIE-HA* and its transformation into *fie-11*

A 4.1-kb *FERTILIZATION-INDEPENDENT ENDOSPERM* (*FIE*) genomic region without the stop codon was fused to 6xHA and cloned into the P1 position of the pAGM4723 vector in the Golden Gate system; Basta resistance was cloned into the P2 position in this vector (Engler *et al.* 2014). The primers used in cloning are listed in Supplementary Table 2. TL 3.31 was crossed to *fie-11* heterozygotes (genotyped using the *fie-11* F and *fie-*11 R1 primers) and the *fie-11* heterozygotes from this cross were selected for further analysis. Moderately fluorescent seeds from the *fie-11* heterozygote’s progeny were planted and the FIE-HA construct was transformed into these plants. Fully developed nonflorescent seeds from the T0 population were planted and single T-DNA insertion lines were selected from the T2 generation by Basta selection. Plants that had 2 copies of *fie -11* and *FIE-HA* were identified by quantitative PCR using a Bio-Rad CFX 96 real-time System.

## Results

**Fig. 1.**
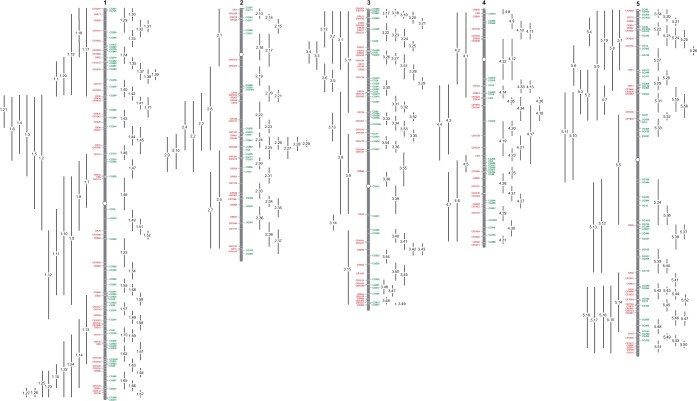
Location of short-interval (right) and long-interval (left) TLs on the chromosomes of the Columbia accession of *Arabidopsis thaliana*.

TLs were generated from a collection of sequenced *pNAP::eGFP* and *pNAP::DsRed* T-DNA insertions, using the approach described in Wu *et al.* (2015) and summarized the *Materials and Methods*. The chromosomal position of these TLs is illustrated schematically in Fig. 1, along with the locations of the TLs described previously. The insertion sites of the transgenes used in the construction of the new lines, and the physical and recombination distance between them are presented in Table 1 and Supplementary Table 1. Although these recombination distances are sometimes based on relatively few individuals, they are of interest because they represent the frequency of recombination across the entire genome within an inbred background. On average, the physical distance between transgenes was 1.2 Mb, and the recombination distance was 6.9 cM. This yields an average recombination rate of 5.6 cM/Mb (Table 1), which is higher than has been observed in crosses between different accessions (Singer *et al.* 2006; Salomé *et al.* 2012). A few lines (e.g. TL1.55, TL2.20) displayed an unusually high rate of recombination whereas others displayed an unusually low rate of recombination compared to other lines in the same region of the genome. Although TLs with unusually low rates of recombination could reflect the actual rate of recombination in this region, this phenomenon could also be due to small chromosomal rearrangements associated with one or both of the transgenes in the TL. Transgenes associated with reciprocal translocations or large inversions were eliminated by screening T1 plants for semisterility, but this approach cannot detect small rearrangements.

**Table 1.** Genomic location and recombination distance between the *pNAP::eGFP* (CG) and *pNAP::DsRed* (CR) insertions in short-interval TLs.

ABRC ID	Line #	Upper marker	Upper marker position	Lower marker	Lower marker position	Length of interval (nt)	Progeny of test cross (Col × dsRed/GFP)					cM	cM/mB
							dsRed	GFP	dsRed + GFP	Nonfluor.	Total		
Chr. 1													
CS72863	1.29	CR1297	77,685	CG308	1,882,501	1,804,816	147	151	19	44	361	19.1	10.6
CS72864	1.30	CG427	92,431	CR924	995,883	903,452	196	194	21	12	423	7.8	8.6
CS72865	1.31	CR924	995,883	CG308	1,882,501	886,618	88	89	3	9	189	6.4	7.2
CS72866	1.32	CR924	995,883	CG246	2,326,356	1,330,473	34	29	3	5	71	11.3	8.5
CS72867	1.33	CR1161	1,778,732	CG945	3,030,311	1,251,579	117	125	7	15	264	8.3	6.6
CS72868	1.34	CG945	3,030,311	CR1312	4,270,245	1,239,934	108	108	8	8	232	6.8	5.5
CS72869	1.35	CR62	3,852,828	CG384	4,767,960	915,132	123	126	14	7	270	7.8	8.5
CS72870	1.37	CR1312	4,270,245	CG538	5,608,046	1,337,801	82	76	1	4	163	3.1	2.3
CS72871	1.38	CG384	4,767,960	CR710	5,897,006	1,129,046	87	88	9	3	187	6.4	5.7
CS72872	1.39	CR1078	4,777,894	CG538	5,608,046	830,152	103	107	4	2	216	2.8	3.4
CS72873	1.40	CG538	5,608,046	CR30	6,899,263	1,291,217	151	166	11	9	337	5.9	4.6
CS72875	1.42	CG926	6,122,779	CR1416	7,179,633	1,056,893	214	196	1	3	414	1.0	0.7
CS72874	1.41	CR30	6,899,263	CG698	7,931,648	1,032,385	156	137	11	8	312	6.1	5.9
CS73210	1.71	CR434	6,995,077	CG298	8,534,178	1,539,101	118	118	15	11	262	9.9	6.4
CS72876	1.43	CG698	7,931,648	CR94	9,321,582	1,389,934	197	176	12	19	404	7.7	5.5
CS72877	1.44	CG914	9,171,797	CR75	10,678,341	1,506,544	76	77	6	15	174	12.1	8.0
CS72878	1.45	CR94	9,321,582	CG712	11,325,954	2,004,372	115	108	7	12	242	7.8	3.9
CS72879	1.46	CR75	10,678,341	CG510	11,820,335	1,141,994	120	116	8	4	248	4.8	4.2
CS72880	1.47	CG510	11,820,335	CR1111	13,207,868	1,387,533	108	86	6	7	207	6.3	4.5
CS72881	1.48	CR1111	13,207,868	CG30	15,662,695	2,454,827	155	153	5	2	315	2.2	0.9
CS72882	1.49	CG30	15,662,695	CR78	17,324,946	1,662,251	86	79	7	2	174	5.2	3.1
CS72883	1.51	CG514	16,346,787	CR1348	17,618,718	1,271,931	96	123	9	4	232	5.6	4.4
CS72884	1.52	CR78	17,324,946	CG529	17,952,797	627,851	141	169	6	6	322	3.7	5.9
CS72885	1.53	CR894	17,885,687	CG669	20,100,577	2,214,890	175	140	20	3	338	6.8	3.1
CS72886	1.54	CR1002	19,802,910	CG289	21,089,182	1,286,272	90	96	6	10	202	7.9	6.1
CS72887	1.55	CG289	21,089,182	CR864	22,097,069	1,007,887	132	143	37	38	350	21.4	21.2
CS72899	1.68	CG505	21,509,119	CR54	22,359,854	850,735	101	82	5	2	190	3.6	4.2
CS72888	1.56	CR864	22,097,069	CG36	23,112,417	1,015,348	115	122	8	7	252	5.9	5.8
CS72889	1.57	CG630	22,923,792	CR939	23,874,536	950,744	114	113	8	8	243	6.6	6.9
CS72900	1.69	CG355	23,494,135	CR1140	24,537,026	1,042,891	161	144	9	7	321	5.0	4.8
CS72890	1.58	CR939	23,874,536	CG47	25,044,965	1,170,429	88	82	11	11	192	11.5	9.8
CS72901	1.70	CR613	24,771,463	CG578	25,878,292	1,106,829	72	71	4	6	321	6.5	5.9
CS72891	1.59	CG47	25,044,965	CR776	25,953,619	908,654	158	143	8	12	321	6.2	6.8
CS72892	1.61	CR776	25,953,619	CG1030	27,308,421	1,354,802	106	109	11	8	234	8.1	6.0
CS72893	1.62	CG439	26,180,908	CR1356	27,484,048	1,303,140	95	104	10	5	214	7.0	5.4
CS72894	1.63	CG1030	27,308,421	CR905	28,213,615	905,194	97	96	4	12	209	7.6	8.4
CS72895	1.64	CR1155	27,990,531	CG549	28,643,159	652,628	57	67	2	5	131	5.3	8.1
CS72896	1.65	CR905	28,213,615	CG915	29,596,565	1,382,950	148	141	12	20	321	10.0	7.2
CS72897	1.66	CG857	28,957,556	CR1112	29,747,473	789,917	162	132	11	6	311	5.5	7.0
CS72898	1.67	CR1112	29,747,473	CG394	30,273,249	525,776	121	131	5	5	262	3.8	7.2
Chr. 2													
CS72902	2.13	CG591	2,114	CR140	858,680	856,566	128	85	18	11	242	11.9	13.9
CS72903	2.14	CR1028	444,653	CG977	968,026	523,373	115	118	9	7	249	6.4	12.2
CS72904	2.15	CR140	858,680	CG531	1,989,262	1,130,582	169	182	9	18	378	7.1	6.3
CS72905	2.16	CG531	1,989,262	CR1014	4,022,387	2,033,125	186	179	9	12	386	5.4	2.7
CS72906	2.17	CG531	1,989,262	CR1047	4,525,187	2,535,925	117	103	5	7	232	5.4	2.1
CS72907	2.19	CR1047	4,525,187	CG559	6,051,986	1,526,799	115	120	8	10	253	7.1	4.7
CS72908	2.20	CG559	6,051,986	CR896	7,229,424	1,177,438	106	113	17	13	249	12.0	10.2
CS72909	2.21	CG1024	6,262,040	CR13	7,242,222	980,182	113	104	5	8	230	5.6	5.7
CS72910	2.22	CR13	7,242,222	CG635	9,379,120	2,136,898	138	170	11	11	330	6.7	3.1
CS72911	2.23	CR128	8,677,163	CG46	9,738,512	1,061,349	162	139	6	3	310	2.9	2.7
CS72912	2.24	CR1147	9,656,849	CG882	10,781,018	1,124,169	180	177	10	14	381	6.2	5.5
CS72913	2.25	CR441	9,976,629	CG515	11,728,603	1,751,974	110	136	13	4	263	6.5	3.7
CS72914	2.26	CR441	9,976,629	CG6	11,048,948	1,072,319	276	281	14	10	581	4.1	3.8
CS72915	2.27	CR640	10,017,780	CG515	11,728,603	1,710,823	269	243	17	6	535	4.2	2.5
CS72916	2.28	CR640	10,017,780	CG6	11,048,948	1,031,168	258	295	13	17	583	5.1	4.9
CS72917	2.29	CG601	10,291,848	CR1026	10,807,952	516,104	128	136	4	7	275	4.0	7.8
CS72918	2.30	CG771	11,608,725	CR929	12,506,436	897,711	152	145	9	10	316	6.0	6.7
CS72919	2.31	CG866	12,387,640	CR1124	13,622,737	1,235,097	83	95	3	1	182	2.2	1.8
CS72920	2.33	CR1124	13,622,737	CG405	14,782,362	1,159,625	157	125	5	9	296	4.7	4.1
CS72921	2.34	CR1381	14,566,497	CG637	15,436,302	869,805	103	93	6	10	212	7.6	8.7
CS72922	2.35	CG405	14,782,362	CR841	16,251,113	1,468,751	191	225	24	18	458	9.2	6.3
CS72923	2.36	CG637	15,436,302	CR1117	17,170,719	1,734,417	141	143	8	9	302	5.6	3.2
CS72925	2.38	CG951	16,663,782	CR1145	18,556,060	1,892,278	167	141	24	28	360	14.4	7.6
CS72924	2.37	CR1117	17,170,719	CG404	19,103,853	1,933,134	75	83	3	4	165	4.2	2.2
Chr. 3													
CS72926	3.17	CG465	45,383	CR1110	707,793	662,410	152	145	14	11	322	7.8	11.8
CS72927	3.18	CG465	45,383	CR921	672,397	627,014	71	66	3	7	147	6.8	10.8
CS72928	3.19	CR1118	90,264	CG834	738,368	648,104	98	93	2	3	196	2.6	4.0
CS72929	3.20	CG211	130,439	CR921	672,397	541,958	129	115	12	13	269	9.3	17.2
CS72930	3.21	CR921	672,397	CG446	1,593,406	921,009	111	109	9	15	244	9.8	10.6
CS72931	3.22	CR92	1,171,464	CG50	2,528,742	1,357,278	98	107	5	13	223	8.1	6.0
CS72932	3.23	CR627	1,496,828	CG50	2,528,742	1,031,914	85	89	10	4	188	7.4	7.2
CS72933	3.24	CG446	1,593,406	CR631	3,025,689	1,432,283	89	95	9	9	202	8.9	6.2
CS72934	3.25	CG50	2,528,742	CR352	3,300,254	771,512	208	201	5	8	422	3.1	4.0
CS72935	3.26	CG21	3,280,460	CR73	4,330,342	1,049,882	209	196	5	10	420	3.6	3.4
CS72936	3.27	CG919	3,365,285	CR73	4,330,342	965,057	185	198	3	14	400	4.2	4.4
CS73211	3.51	CR1420	3,493,180	CG310	5,440,370	1,947,190	86	84	11	6	187	9.1	4.7
CS72937	3.28	CR73	4,330,342	CG24	5,603,768	1,273,426	116	122	5	6	249	4.4	3.5
CS72938	3.29	CR1120	5,351,946	CG991	6,264,102	912,156	67	75	2	6	150	5.3	5.8
CS72939	3.30	CG24	5,603,768	CR1045	6,761,383	1,157,615	125	106	5	3	239	3.3	2.9
CS72940	3.31	CR1045	6,761,383	CG43	8,017,809	1,256,426	139	135	7	18	299	8.4	6.7
CS72941	3.32	CG521	7,256,475	CR729	8,072,294	815,819	99	106	2	6	213	3.7	4.5
CS73212	3.52	CG43	8,017,809	CR1267	8,743,242	725,433	118	153	11	11	293	7.5	10.4
CS72942	3.33	CR729	8,072,294	CG1026	8,986,977	914,683	129	138	9	10	286	6.6	7.2
CS72943	3.34	CG654	8,298,846	CR1076	9,417,490	1,118,644	146	168	15	23	352	10.8	9.7
CS73213	3.53	CR1267	8,743,242	CG931	9,946,151	1,202,909	154	131	14	12	311	8.4	6.9
CS72944	3.35	CR1076	9,417,490	CG906	10,318,203	900,713	127	130	4	10	271	5.2	5.8
CS73214	3.54	CG931	9,946,151	CR1004	10,960,795	1,014,644	106	114	3	7	230	4.4	4.3
CS72945	3.36	CG906	10,318,203	CR1121	11,132,015	813,812	210	210	12	14	446	5.8	7.1
CS73215	3.55	CR1121	11,132,015	CG912	13,809,565	2,677,550	140	156	4	10	310	4.5	1.7
CS72946	3.38	CR880	12,643,363	CG912	13,809,565	1,166,202	419	451	1	4	875	5.7	4.9
CS72947	3.39	CG912	13,809,565	CR55	15,980,483	2,170,918	115	126	5	6	252	4.3	2.0
CS72948	3.40	CG329	17,200,480	CR1437	18,145,873	945,393	157	174	9	5	345	4.0	4.2
CS72949	3.41	CG520	17,452,950	CR895	18,751,094	1,298,144	178	145	6	10	339	4.7	3.6
CS72950	3.42	CR1041	18,080,606	CG925	19,199,320	1,118,714	94	106	6	7	213	6.1	5.5
CS72951	3.43	CR1437	18,145,873	CG925	19,199,320	1,053,447	155	177	15	5	352	5.7	5.4
CS72952	3.44	CR895	18,751,094	CG650	19,802,350	1,051,256	135	133	9	9	286	6.3	6.0
CS72958	3.50	CG650	19,802,350	CR1015	21,130,575	1,328,225	84	83	4	7	178	6.2	4.7
CS72953	3.45	CR903	19,898,699	CG691	21,471,664	1,572,965	110	125	10	7	252	6.7	4.3
CS72954	3.46	CG932	21,049,086	CR865	22,219,925	1,170,839	163	181	14	14	372	7.5	6.4
CS72955	3.47	CG691	21,471,664	CR1154	22,406,157	934,493	112	127	9	10	258	7.4	7.9
CS72956	3.48	CR1154	22,406,157	CG927	22,909,706	503,549	92	99	11	3	205	6.8	13.5
CS72957	3.49	CG913	22,853,491	CR389	22,974,799	121,308	143	146	2	4	295	2.0	16.5
Chr. 4													
CS72959	4.8	CR943	85,561	CG873	376,780	291,219	171	145	10	15	341	7.3	25.1
CS72960	4.9	CG873	376,780	CR1130	1,586,387	1,209,607	146	148	18	23	335	12.2	10.1
CS72961	4.10	CG490	998,987	CR1628	2,381,455	1,382,468	177	201	12	11	401	5.7	4.1
CS72962	4.11	CG509	1,138,245	CR1628	2,381,455	1,243,210	174	151	13	10	348	6.6	5.3
CS72963	4.12	CR1628	2,381,455	CG350	5,503,814	3,122,359	85	63	11	11	170	12.9	4.1
CS72978	4.32	CR1628	2,381,455	CG910	5,452,727	3,071,272	126	129	15	10	280	8.9	2.9
CS72979	4.33	CG910	5,452,727	CR861	6,573,205	1,120,478	95	124	10	4	233	6.0	5.4
CS72964	4.13	CG350	5,503,814	CR861	6,573,205	1,069,391	138	128	12	11	289	8.0	7.5
CS72965	4.14	CR142	6,030,331	CG8	6,955,655	925,324	110	123	7	8	248	6.0	6.5
CS73216	4.34	CR1422	6,895,541	CG916	8,750,678	1,855,137	165	167	15	20	367	9.5	5.1
CS73217	4.35	CG8	6,955,655	CR1660	7,522,130	566,475	118	130	3	6	257	3.5	6.2
CS73218	4.36	CG8	6,955,655	CR1305	7,544,169	588,514	95	104	9	5	213	6.6	11.2
CS72966	4.16	CR1660	7,522,130	CG916	8,750,678	1,228,548	78	85	3	4	170	4.1	3.3
CS72967	4.17	CR1305	7,544,169	CG513	12,137,086	4,592,917	229	256	51	61	597	18.8	4.1
CS72968	4.19	CG916	8,750,678	CR3	11,440,981	2,690,303	84	69	10	6	169	9.5	3.5
CS72969	4.20	CG916	8,750,678	CR1108	9,875,815	1,125,137	117	125	5	9	256	5.5	4.9
CS72970	4.21	CR1108	9,875,815	CG2	11,872,893	1,997,078	79	93	8	12	192	10.4	5.2
CS72971	4.23	CR1279	10,825,656	CG2	11,872,893	1,047,237	132	149	6	5	292	3.8	3.6
CS72972	4.25	CG262	12,337,463	CR800	13,192,747	855,284	133	117	7	3	260	3.8	4.4
CS72973	4.26	CG29	12,727,651	CR1394	14,380,651	1,653,000	118	131	6	10	265	6.0	3.6
CS73219	4.37	CR1107	13,813,834	CG864	14,954,316	1,140,482	163	149	6	7	325	4.0	3.5
CS72974	4.27	CR1394	14,380,651	CG507	15,542,832	1,162,181	193	200	13	10	416	5.5	4.7
CS72975	4.29	CR1452	15,399,547	CG1020	16,460,866	1,061,319	141	138	7	9	295	5.4	5.1
CS72976	4.30	CG1020	16,460,866	CR1092	17,743,599	1,282,733	139	146	11	18	314	9.2	7.2
CS72977	4.31	CG753	17,649,518	CR1451	18,296,565	647,047	115	104	7	8	234	6.4	9.9
Chr. 5													
CS73012	5.54	CG45	86,161	CR775	657,523	571,362	149	143	8	11	311	6.1	10.7
CS72980	5.20	CG45	86,161	CR906	992,521	906,360	138	125	19	22	304	13.5	14.9
CS72981	5.21	CR775	657,523	CG49	1,307,945	650,422	114	90	6	9	309	7.4	11.4
CS72982	5.22	CR906	992,521	CG345	2,076,237	1,083,716	160	176	14	14	364	7.7	7.1
CS72983	5.23	CG49	1,307,945	CR1156	2,874,982	1,567,037	106	96	15	15	232	12.9	8.2
CS72984	5.24	CR947	1,483,635	CG892	3,078,592	1,594,957	137	149	9	14	309	7.4	4.6
CS72985	5.25	CR1597	1,684,117	CG892	3,078,592	1,394,475	117	141	13	11	282	8.5	6.1
CS72986	5.26	CR1156	2,874,982	CG738	3,592,092	717,110	132	137	6	7	282	4.6	6.4
CS72987	5.27	CG892	3,078,592	CR41	4,735,563	1,656,971	135	137	8	13	293	7.2	4.3
CS72988	5.28	CR41	4,735,563	CG719	5,915,491	1,179,928	165	144	8	6	323	4.3	3.6
CS72989	5.29	CG767	5,240,098	CR1645	6,274,480	1,034,382	96	84	1	5	186	3.2	3.1
CS73220	5.55	CG495	6,043,335	CR1336	7,987,368	1,944,033	140	135	11	19	305	9.8	5.1
CS72990	5.31	CG855	6,585,030	CR1336	7,987,368	1,402,338	166	183	11	11	371	5.9	4.2
CS73221	5.56	CG855	6,585,030	CR1635	8,515,260	1,930,230	149	146	14	16	325	9.2	4.8
CS72991	5.32	CR1336	7,987,368	CG4	8,647,153	659,785	165	177	3	9	354	3.5	5.3
CS72992	5.33	CR1635	8,515,260	CG597	9,890,330	1,375,070	202	188	16	17	423	7.8	5.7
CS72993	5.34	CG358	13,229,304	CR29	16,780,239	3,550,935	163	155	34	39	391	18.7	5.3
CS72994	5.36	CG16	15,254,136	CR29	16,780,239	1,526,103	199	207	15	21	442	8.1	5.3
CS72995	5.37	CR29	16,780,239	CG621	17,850,058	1,069,819	304	318	18	19	659	5.6	5.2
CS72996	5.38	CR29	16,780,239	CG518	18,678,795	1,898,556	163	155	34	39	391	18.7	9.8
CS72997	5.39	CG518	18,678,795	CR39	20,491,443	1,812,648	203	203	15	18	439	7.5	4.1
CS72998	5.40	CG605	19,327,782	CR39	20,491,443	1,163,661	94	98	5	1	198	3.0	2.6
CS72999	5.41	CR39	20,491,443	CG326	21,542,180	1,050,737	99	91	3	5	198	4.0	3.8
CS73000	5.42	CR983	21,542,180	CG720	22,522,561	980,381	167	194	15	12	388	6.9	7.0
CS73001	5.43	CG326	21,542,180	CR1603	22,170,459	628,279	178	183	6	13	380	5.0	8.0
CS73002	5.44	CR58	21,803,754	CG19	22,594,347	790,593	48	56	2	2	108	3.7	4.7
CS73010	5.52	CR1603	22,170,459	CG354	23,038,968	868,509	232	216	6	6	460	2.6	3.0
CS73003	5.45	CG19	22,594,347	CR1081	23,501,247	906,900	213	213	13	10	449	5.1	5.6
CS73004	5.46	CR1114	23,415,403	CG608	24,596,395	1,180,992	106	120	2	2	230	1.7	1.4
CS73005	5.47	CR1081	23,501,247	CG608	24,596,395	1,095,148	209	206	8	4	427	2.8	2.6
CS73006	5.48	CG668	24,115,449	CR59	25,204,540	1,089,091	153	135	11	9	308	6.5	6.0
CS73007	5.49	CR59	25,204,540	CG769	25,742,640	538,100	121	113	6	6	246	4.9	9.1
CS73011	5.53	CG454	25,204,540	CR911	26,171,385	966,845	129	127	17	9	282	9.2	9.5
CS73008	5.50	CG769	25,742,640	CR106	26,333,257	590,617	165	123	12	15	315	8.6	14.6
CS73009	5.51	CG769	25,742,640	CR715	26,684,741	942,101	171	205	28	23	427	11.9	12.6

### Using a TL to genotype seeds

**Fig. 2.**
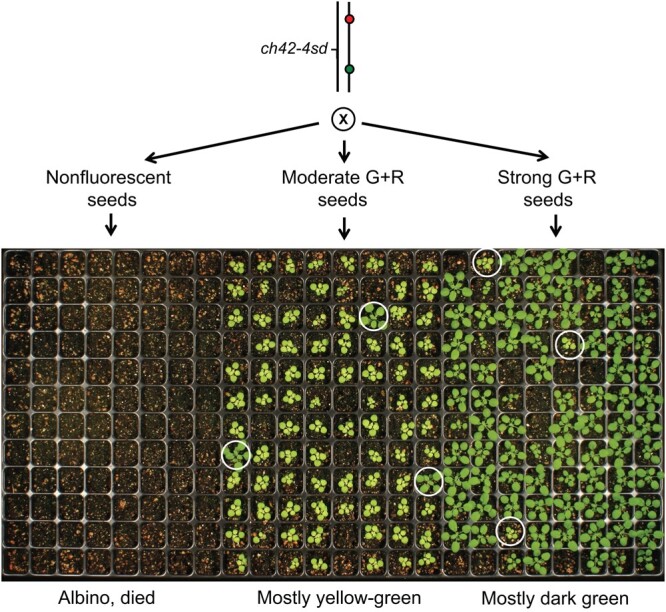
Self-progeny of a *ch42-4sd*/TL4.22 plant, planted according to the seed fluorescence phenotype. Nonfluorescent seeds are expected to be predominantly *ch42-4sd/ch42-4sd* and albino, moderately fluorescent seeds are expected to be predominantly *ch42-4sd*/TL4.22 and yellow-green, and strongly fluorescent seeds are expected to be predominantly TL4.22/TL4.22 and dark green. Seedlings that do not conform to the expected phenotype are circled.

It is often important to be able to distinguish different genotypes in a segregating population. This is particularly important if one wants to compare the phenotype of mutants with weak or poorly penetrant phenotypes with their wild-type siblings. In *Arabidopsis*, this is typically done by performing allele-specific PCR on plants after germination. To demonstrate that TLs can be used to perform this task, we took advantage of a semidominant allele of *CHLORINA 42* (*ch42-4sd*) that has a yellow-green phenotype when heterozygous and is albino when homozygous. Plants heterozygous for *ch42-4sd* were crossed to TL4.22, a line that spans the *CH42* locus and contains transgenes located 1.8 Mb from each other. Yellow-green progeny from this cross were allowed to self-fertilize, and nonfluorescent, moderately fluorescent red and green, and strongly fluorescent red and green seed were selected (Fig. 2). One hundred percent of the nonfluorescent seeds produced albino seedlings, 97% of the moderately fluorescent seeds produced yellow-green seeds, and 97% of the strongly fluorescent seeds produced dark-green seedlings. Thus, TLs are a reliable tool for determining the genotype of a seed prior to planting.

### Using TLs for phenotypic analysis

Determining the phenotype of a mutation in a known gene can be problematic if the mutation has a weak phenotype or is present in a genetic background different from the one that is commonly used in a laboratory. The latter problem can occur if the mutation was generated in a different accession (e.g. Ler instead of Col), or is in a mutagenized background, which may contain other linked and unlinked mutations. One way to get around this problem is to compare the phenotype of mutant and wild-type siblings in the same family. On average, these individuals will share the same background, even if this background is not completely uniform.

**Fig. 3.**
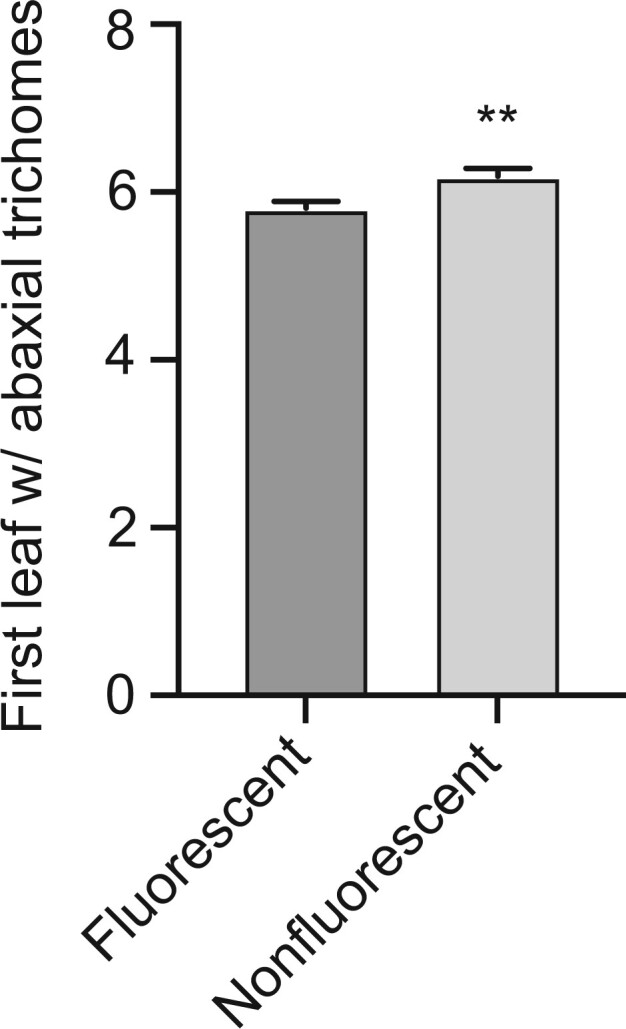
The first leaf with abaxial trichomes in the self-progeny of a *myb75-1*/TL1.55 plant, planted according to seed fluorescence phenotype. Nonfluorescent seeds are expected to be predominantly *myb75-1/myb75-1* and moderately fluorescent seeds are expected to be predominantly *myb75-1*/TL1.55. Error bars = SEM; ** = *P* < 0.01, 2-tailed Student’s *t*-test, n > 45.

To test the usefulness of TLs for comparing the phenotype of genetically different siblings, we examined the phenotype of *myb75-1* in a moderately heterogeneous genetic background. This mutation was of particular interest because it has been reported to delay the transition from juvenile to adult growth (vegetative phase change) under short days, but not long days (Meng *et al.* 2021). *myb75-1* is a Ds-induced allele originally isolated in the Nössen accession (Teng *et al.* 2005). We crossed *myb75-1* to Col 5 times and then crossed these *myb75-1* plants to TL1.55, a TL that spans a 1-Mb interval containing the *MYB75* locus. Nonfluorescent and moderately fluorescent seeds produced by TL1.55/*myb75-1* plants were grown separately in the same flat under long-day conditions. As shown in Fig. 3, nonfluorescent seeds had slightly, but significantly, later abaxial trichomes than their fluorescent siblings. This result demonstrates that *myb75-1* delays vegetative phase change under both long-day and short-day conditions.

### Using TLs to map a novel mutation

In addition to being useful for tracking the segregation of known genes, TLs can also be used to map novel mutations or natural variants. As an example, we used a set of long-interval TLs (Wu *et al.* 2015) to map a novel juvenilized mutant present in a transgenic stock of Col obtained from the *Arabidopsis* Biological Resource Center. This mutation was unlinked to the known T-DNA insertion in this line, and a subsequent “mapping by bulked genome sequencing” approach failed to identify a polymorphism that segregated with this mutation. As an alternative approach, we crossed the mutant to a set of long-interval TLs spanning the *Arabidopsis* genome, and determined if the mutant phenotype was linked to markers in these lines by planting 12–15 nonfluorescent and a similar number of strongly fluorescent seeds from the F2 progeny of these crosses. In most cases, wild-type and mutant seedlings were present in both the fluorescent and nonfluorescent seed classes in approximately equal ratios, with wild-type seedlings typically being more abundant than mutant seedlings (the expected ratio is 3/4 wild type:1/4 mutant for an unlinked TL; Table 2). In contrast, all of the fluorescent seeds from the cross to TL4.6 had a wild-type phenotype, and almost all of the nonfluorescent seeds had a mutant phenotype. This result suggests that the mutation is located within, or close to, the segment marked by TL4.6.

**Table 2.** Mapping a recessive mutation using a genome-wide set of long-interval TLs.

Seed phenotype	Number of WT: mutant progeny produced by TL/mutant plants											
	TL1.9	TL1.13	TL2.5	TL3.8	TL3.5	TL3.11	TL4.1	TL4.4	TL4.6	TL5.8	TL5.12	TL5.14
Fluorescent	5:1	9:3	11:3	9:2	9:1	9:3	9:2	8:1	**12:0**	11:2	5:6	11:4
Nonfluorescent	5:2	10:2	9:3	9:2	4:3	8:3	8:4	4:7	**1:12** ^*^	11:1	9:4	10:4

^*^ The high frequency of mutant seedlings among nonfluorescent seeds indicates that the mutation is within, or near, this TL.

### Using TLs to rescue a lethal or sterile mutation with a transgene

It is often desirable to generate a transgenic line carrying an epitope-tagged protein (e.g. a reporter protein, or an affinity-tagged protein). Ideally, the epitope-tagged construct should be transformed into a line carrying a null mutation of the corresponding gene to demonstrate that the construct is able to rescue the mutant phenotype and ensure that the only functional copy of the protein is the version produced by the transgene. This is straightforward when the homozygous mutant is viable and fully fertile, and can therefore be directly transformed with the construct. It is more difficult when plants homozygous for the mutation are lethal or sterile. In this case, the simplest approach is to transform the construct into wild-type plants and then introduce the construct into a homozygous mutant background by several generations of crossing. This is a time- and space-consuming process and requires a considerable amount of molecular genotyping.

**Fig. 4.**
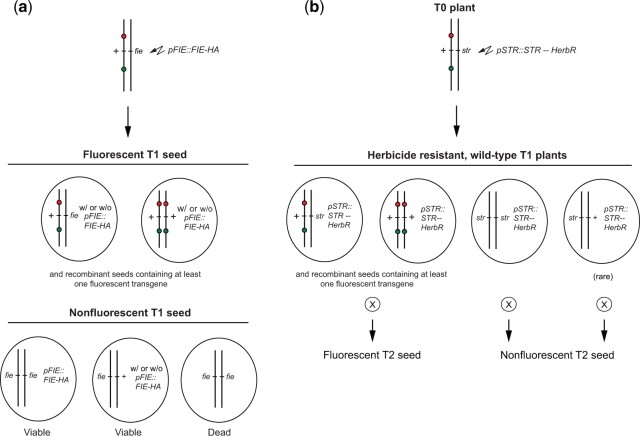
Rescuing lethal or sterile mutations with a transgene. a) Moderately fluorescent seeds from a *fie-11*/TL3.31 plant were selected and the resulting plants were transformed with a *pFIE-HA BaR* T-DNA construct by floral dipping. The expected genotypes of the nonfluorescent seeds from these plants are shown. Fully formed (i.e. viable) seeds will either be homozygous for *fie-11* and hemizygous for *pFIE-HA BaR*, or be heterozygous for *fie-11* as a result of double recombination within the interval marked by TL3.31. Given the relatively low frequency of transformation, this latter class of seeds will probably lack *pFIE-HA BaR*. b) Using a TL to rescue a seedling lethal or sterile mutant with a transgene. This approach is useful if the mutation does not affect seed morphology or viability, making it impossible to distinguish transgenic from nonransgenic seeds prior to planting.

TLs facilitate this process by providing a convenient method for identifying plants homozygous and heterozygous for lethal/sterile mutations. As an example, we used a TL to generate a line carrying an HA-tagged version of FIE, an essential component of the PRC2 complex (Ohad *et al.* 1999). Plants homozygous for the null allele, *fie-11*, are embryonic lethal, making it necessary to maintain this mutation in heterozygous condition (Guitton *et al.* 2004). We generated a balanced stock by crossing *fie-11*/+ plants to TL3.31 and identified the *fie-11*/TL3.31 plants from this cross using PCR primers specific for *fie-11*. We then selected moderately fluorescent green and red seed from these plants (which were expected to be *fie-11*/TL3.31), and used floral dipping to transform these plants with a T-DNA construct containing *pFIE::FIE-HA* and Basta-resistance as a selectable marker (Fig. 4a). Approximately 75% of the T1 seeds resulting from this experiment were fluorescent and were expected to either be heterozygous for *fie-11* or homozygous wild type (Fig. 4). Most of the remaining seeds were shriveled and dead, as expected because *fie-11* is lethal in homozygous condition. We also identified 14 nonfluorescent, viable seeds (Fig. 4a). These T1 seeds were planted, and their progeny were screened for Basta-resistance. Three of these families segregated ¼ dead seed and were sensitive to Basta indicating that the parental T1 seeds were heterozygous for *fie-11* and not transgenic. These seeds were likely the result of double recombination within the marked segment which transferred the wild-type allele of *FIE* to an unmarked chromosome. The remaining 11 families segregated ¼ or less dead seeds, and all the viable seeds were resistant to Basta. Six of the families had very few dead seed, suggesting that they contained multiple, independent, *pFIE::FIE-HA* insertions. Five families had ¼ dead seed, implying that the parental T1 seed was homozygous for *fie-11* and heterozygous for a single *pFIE::FIE-HA* insertion. Seedlings from at least 6 plants in each of these single-insertion families were treated with Basta to identify families homozygous for the transgene. Subsequent quantitative PCR analysis of the dosage of *fie-11* and FIE-HA (primer sequences in Supplementary Table 2) demonstrated that these lines were indeed *fie-11/fie-11pFIE::FIE-HA*/*pFIE::HA*.

An alternative approach is useful for lethal or sterile mutations whose seeds are phenotypically normal (Fig. 4b). In this case, it is impossible to identify which nonfluorescent seeds contain the transgene of interest because transgenic seeds are visually indistinguishable from nontrangenic seeds. Transgenic plants can be identified after germination using a selectable marker (e.g. Basta resistance) present on the transgene, but if the transformation efficiency is relatively low this will require selecting and planting a large number of nonfluorescent seeds, which is tedious. Alternatively, one can screen a large number of T1 plants for the presence of the selectable marker and then screen the T2 seeds produced by these plants for seed fluorescence. Approximately 1/4 of the plants containing the selectable marker should have nonfluorescent seeds and be homozygous for the lethal/sterile mutation.

### Using TLs to separate tightly linked mutations

Next-generation sequencing has revealed that many induced mutations are associated with linked second-site mutations (Schneeberger *et al.* 2009; Enders *et al.* 2015; Thole and Strader 2015; Lup *et al.* 2021). Determining whether the phenotype of these lines is attributable to one or more of these linked mutations can be problematic if the mutations are so tightly linked that they do not readily segregate from each other. We identified an EMS-induced late vegetative phase change mutant that contained missense mutations in 2 members of the PRC1 complex, *VAL1* and *AtBMI1A* (Molitor and Shen 2013; Merini and Calonje 2015). Null alleles of each gene have a much weaker effect on vegetative phase change than this double mutant, so we assumed that the relatively strong phenotype of the double mutant was attributable to the combined effects of these missense mutations. One way to test this hypothesis is to separate these mutations by recombination and examine their individual phenotypes. However, *VAL1* and *AtBMI1A* are located only 45.5 kb from each other on chromosome 2, making this an extremely time- and resource-intensive experiment to perform by molecular genotyping.

**Fig. 5.**
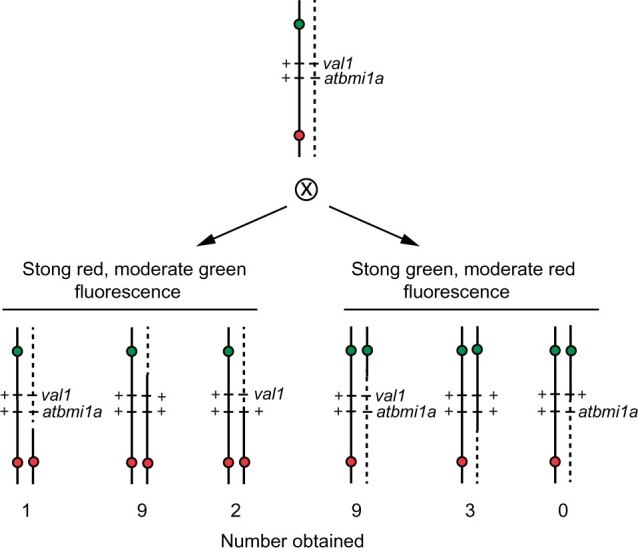
Using a TL to separate closely linked mutations. F2 seeds from a *val1 bmi1a*/TL2.31 plant were screened for seeds that displayed strong red and moderate green fluorescence, or strong green and moderate red fluorescence. The genotype of these seeds was determined by examining the molecular genotype of their F2 progeny, using allele-specific PCR primers.

We selected 12 strong red, moderate green, and 12 strong green, moderate red seeds from the F2 progeny of plants heterozygous for TL2.31 and *val1 atbmi1a* (Fig. 5). Because these genes are located ca. 600 Mb from both the *pNAP::eGFP* and *pNAP::DsRed* transgenes, we expected approximately equal numbers of *val1 atbmi1a/++* and ++/++ recombinants and a much smaller number of *val1* +/++ and + *atbmi1a*/++ recombinants. Consistent with this expectation, we obtained only 2 *val1* +/++ recombinants and no + *atbmi1a*/++ recombinants. Unexpectedly, we found that the number of recombinants between *pNAP::eGFP* and *val1* was 3 times greater than the number of recombinants between *pNAP::DsRed* and *val1* (18 vs 6). This was surprising because *val1 atbmi1a* are equidistant from these transgenes. This suggests that the *pNAP::DsRed* insertion in TL2.31 (CR1124) is associated with a small rearrangement that suppresses recombination. This hypothesis is supported by the observation that the cM/Mb ratio in TL2.31 is significantly less than the ratio in a different line (TL2.32) that spans the same region (Table 1).

Subsequent analysis of the phenotype of the *val1* single mutant (*val1-5sd*) revealed that it is semidominant (Fouracre *et al.* 2021), and that its phenotype is identical to the phenotype of the original *val1-5sd atbmi1a* double mutant. This result suggests that—in contrast to our initial hypothesis—the missense mutation in *AtBMI1A* makes no contribution to the phenotype of the double mutant and may be phenotypically silent. It also suggests that *val1-5sd* interferes with the function of VAL2 or other members of the PRC1 complex, as null alleles of *VAL1* have a weaker phenotype than *val1-5sd* (Fouracre *et al.* 2021).

## Discussion

The value of linked fluorescent transgenes for studies of recombination has been recognized for some time (Melamed-Bessudo *et al.* 2005; Berchowitz and Copenhaver 2008). The utility of such transgenes for following the segregation of chromosome segments in *trans* to the marked chromosome is less well appreciated, but is an equally important feature of these lines. This property relies on the fact that a nonfluorescent chromosome segregating from a doubly marked chromosome will either represent a nonrecombinant chromosome, or a chromosome that has undergone double recombination within the marked interval (Wu *et al.* 2015). The frequency of double recombination decreases with the distance between markers, so it is beneficial to use TLs that define relatively small intervals if the goal is to use the TL as a balancer.

For an interval of 6.9 cM—the average for the TLs described here—the maximum frequency of double recombination is 0.035 × 0.035, or approximately 1/1000 chromosomes, although this number is likely to be significantly lower due to crossover interference (Berchowitz and Copenhaver 2010; Salomé *et al.* 2012). This means that the nonfluorescent chromosomes produced by a plant heterozygous for a short-interval TL are very likely to have the genotype of the non-TL chromosome. Furthermore, for any single nucleotide within this interval (e.g. a mutation or polymorphism), the probability that a nonfluorescent chromosome will carry the parental form of this nucleotide increases the farther the nucleotide is from the midpoint of the marked interval. This is because the probability that double recombination will replace the allele on the nonfluorescent chromosome with the allele from the TL decreases as one moves away from the center of the interval; i.e. the closer a gene is to one of the transgenes in a TL, the less susceptible it is to double recombination. Thus, even relatively large TLs can be useful for gene mapping, as we found in the case of a novel phase change mutation that we mapped to the bottom of chromosome 4. Single fluorescent transgenes are also useful for following polymorphisms/mutations in *trans* to the transgene if the polymorphisms/mutations is sufficiently close to the transgene (Wu *et al.* 2015).

Although recombination within a TL is disadvantageous if the TL is being used to follow the segregation of a chromosome in *trans* to the TL, this feature makes it possible to dissect relatively small chromosomal regions. As described here, we were able to recombinationally separate mutations located only 45 kb (ca. 0.03 cM) from each other by visual screening seeds for fluorescence intensity. This process only took a few hours. In contrast, screening the same F2 population for recombinants by PCR would have required hundreds or possibly thousands of quantitative PCR reactions, at considerable time and expense. The ability to rapidly identify and then rescue recombinants in a small chromosomal region is not only useful for dissociating second-site mutations but also facilitates fine mapping of mutations or natural variants that have been localized to specific regions of the genome. It is particularly useful for mapping variants with weak phenotypes because the dosage sensitivity of the fluorescent markers makes it possible to identify plants homozygous for recombinant chromosomes in F2 populations. This facilitates determining which allele of the variant the chromosome possesses because the phenotype of this allele is likely to be strongest when it is homozygous.

An ideal TL is one in which the transgenes are not subject to silencing, are expressed strongly and uniformly in dry seeds, display clear differences in fluorescence intensity in 1 and 2 doses, and are inserted in functionally silent positions in the genome. Unfortunately, this is difficult to achieve. It is easy to produce T-DNA-transformed lines in *Arabidopsis*, but a large number of these lines contain more than one insertion, and many contain a translocation or other type of large rearrangement at the site of the T-DNA insertion (Clark and Krysan 2010; Wu *et al.* 2015). Consequently, only some of the primary transgenic lines are useful for the production of TLs. The existence of small inversions that do not lead to semisterility but which eliminate or reduce recombination is another problem. These are useful if the TL is being used as a balancer, but are problematic if the TL is being used to identify recombination in a defined interval, as was the case in our experiment with the *val1 atbmi1a* double mutant. Finally, there is the problem of transgene silencing. Some of the transgenes we identified silenced after several rounds of propagation, and the frequency of this phenomenon increased for transgenes located close to the centromere. Indeed, it was sometimes necessary to abandon a TL late in the process of generating or amplifying the line because one or both of the markers underwent silencing. Unfortunately, this behavior is typical of transgenes in plants, and only repeated propagation will demonstrate if the transgenes in a TL are sufficiently stable for prolonged use. An expanded collection of *pNAP::eGFP* and *pNAP::DsRed* insertions will enable us to minimize these problems in future collections of TLs. Despite these limitations, the collection of TLs we have generated enable many experiments that are difficult or sometimes impossible to perform, and therefore represent a useful addition to the resources available for genetic analysis in *A. thaliana*.

## Supplementary Material

jkac202_Supplemental_Table_Legends

jkac202_Supplementary_Table_1

jkac202_Supplementary_Table_1

## Data Availability

Short-interval TLs are available as homozygous stocks from the Arabidopsis Biological Resource Center (ABRC), under the stock numbers provided in Table 1. The ABRC stock numbers for the long-interval lines described previously are provided in Wu *et al.* (2015). The transgenes used to construct these lines are available from the ABRC, under the stock numbers provided in Supplementary Table 1. Other genetic stocks described in this article are also available from the ABRC or from the corresponding author. Supplemental material is available at *G3* online.
