# Supplementary material for: Short-interval traffic lines: versatile tools for genetic analysis in Arabidopsis thaliana
Source: G3 (Bethesda). 2022 Aug 26;12(10):jkac202. doi: 10.1093/g3journal/jkac202 (PMC9526051; doi:10.1093/g3journal/jkac202)
Supplement: jkac202_Supplemental_Table_Legends [file jkac202_supplemental_table_legends.docx]

**Supplementary Information**

**Supplementary Table 1.** Genomic location of pNAP::eGFP (CG) and pNAP::DsRed (CR) insertions in transgenic lines of *Arabidopsis thaliana* (Col), and the positions of the transgenes used to construct Traffic Lines.

**Supplementary Table 2.** Primers used in this study.
